# Tracking neuronal marker expression inside living differentiating cells using molecular beacons

**DOI:** 10.3389/fncel.2013.00266

**Published:** 2013-12-19

**Authors:** Mirolyuba Ilieva, Paolo Della Vedova, Ole Hansen, Martin Dufva

**Affiliations:** ^1^Department of Micro- and Nanotechnology, Technical University of DenmarkKgs. Lyngby, Denmark; ^2^Center for Individual Nanoparticle Functionality, Technical University of DenmarkKgs. Lyngby, Denmark

**Keywords:** neural stem cells, differentiation, molecular beacons, gene expression, neuronal marker

## Abstract

Monitoring gene expression is an important tool for elucidating mechanisms of cellular function. In order to monitor gene expression during nerve cell development, molecular beacon (MB) probes targeting markers representing different stages of neuronal differentiation were designed and synthesized as 2'-O-methyl RNA backbone oligonucleotides. MBs were transfected into human mesencephalic cells (LUHMES) using streptolysin-O-based membrane permeabilization. Mathematical modeling, simulations and experiments indicated that MB concentration was equal to the MB in the transfection medium after 10 min transfection. The cells will then each contain about 60,000 MBs. Gene expression was detected at different time points using fluorescence microscopy. *Nestin* and *NeuN* mRNA were expressed in approximately 35% of the LUHMES cells grown in growth medium, and in 80–90% of cells after differentiation. *MAP2* and tyrosine hydroxylase mRNAs were expressed 2 and 3 days post induction of differentiation, respectively. Oct 4 was not detected with MB in these cells and signal was not increased over time suggesting that MB are generally stable inside the cells. The gene expression changes measured using MBs were confirmed using qRT-PCR. These results suggest that MBs are simple to use sensors inside living cell, and particularly useful for studying dynamic gene expression in heterogeneous cell populations.

## Introduction

The ability to detect mRNAs in individual live cells allows investigators to exactly pinpoint when a gene is turned on and off in response to a stimulus. Detecting and measuring gene expression has traditionally been limited to the use of technologies such as DNA microarrays, reverse-transcription polymerase chain reaction (RT-PCR), northern blotting, and fluorescence *in situ* hybridization (FISH), all of which examine the gene expression in lysed or chemically-fixed cell populations. In contrast to these destructive methods, green fluorescent protein labeling (GFP) can be used to track gene expression in living cells. However, GFP and other similar reporter systems cannot measure endogenous mRNA expression in living cells but rely on fusing the GFP gene to the promoter region of interest. GFP/promoter constructs might be integrated into the host genome or be transiently transfected as non-integrating plasmids. Furthermore, the GFP gene and its products (mRNA and proteins, respectively) are not necessarily processed in the same way as the native gene and its products, which can lead to errors in measurement (Lee et al., 2006; Dobek et al., 2011).

Molecular beacon technology was first described in Tyagi and Kramer (1996). Molecular beacons (MBs) are stem-loop forming oligonucleotides with a fluorochrome on one end, and a quencher on the other end. MB recognize its target through the loop and when hybridized displaces the quencher from the fluorochrome. The MBs enables one-step detection of specific nucleic acids in homogeneous solutions (Tyagi and Kramer, 1996). Theoretically, this makes MBs an ideally suitable tool for monitoring gene expression inside living cells on the mRNA level. Despite that, there are far fewer reports describing the use of MBs for monitoring gene expression in living cells compared to the number of reports describing usage of GFP labeling. Bratu et al. (2003) used MBs to visualize the distribution and transport of *oskar* mRNA in Drosophila oocytes. Santangelo et al. (2004) used MBs to analyze the distribution and transport of mRNA in intracellular organelles, and demonstrated that both mRNAs for *GAPDH* and *K-Ras* were localized in the mitochondria. The combination of protein detection with antibodies and mRNA detection with MBs has been used to detect and isolate rare cancer stem cells from populations of normal cells, using fluorescence activated cell sorting (Rhee and Bao, 2009). MBs targeting the *OCT4* mRNA, which is highly expressed in embryonic and cancer stem cells, were introduced into mouse carcinoma cell line without affecting cell function. The MB toward *OCT4* was used to discriminate between undifferentiated and retinoic acid-differentiated cells (Rhee and Bao, 2009). MBs targeting *SOX2* mRNA were used as the sole discriminator to sort mouse embryonic and neural stem cells (Larsson et al., 2012). The isolated *SOX2* mRNA-positive cells formed neurospheres more efficiently than *SOX2* mRNA-negative cells. The clinical and diagnostic utility of MBs was demonstrated in a feasibility study on bladder cancer (Zhao et al., 2010), in which MBs were used to detect survivin mRNA. However, the MB-based assay produced some false positive results, which compromised its immediate use for routine diagnosis. MBs have also been used to monitor expression of two microRNAs (miR-26a and miR-206) during myogenesis (Kang et al., 2011). This study used two MBs with different dyes and quenchers, allowing simultaneous visualization of both miRNAs during myogenesis. Real-time changes in β1-integrin expression in osteoblasts in response to surface modification were tracked with MBs over short periods of time; this study was particularly powerful since changes in mRNA localization were visualized in the same live cells (Lennon et al., 2010). Finally, MBs were used to monitor the temporal gene expression of osteogenic markers, including alkaline phosphatase, type I collagen and osteocalcin during differentiation of adipose-derived stem cells (Desai et al., 2013).

In contrast to hybridization in solution where the physicochemical conditions are simplified, hybridization of MBs to mRNA in living cells is complicated by the formation of secondary structures in the mRNA molecules, RNA-binding proteins, and the degradation of the probes due to enzymes with nuclease activity (Rhee et al., 2008). Software programs such as mFOLD and Beacon Designer can predict the formation of secondary structures so that these sequences can be avoided during the selection of probes in the design process. The prediction of protein binding sites in the target is more complicated due to only limited data existing for RNA-binding proteins (Rhee et al., 2008). Competition with RNA or RNA-binding proteins can result in a significant decrease in signal level due to inefficient hybridization between the MB and the target sequence. To overcome this, many studies have used 2′-O-methyl RNA MBs in order to increase the affinity for the target mRNA (Tsourkas et al., 2002). In addition, a modified backbone is less vulnerable to nuclease activity. However, the formation of double-stranded RNA can lead to RNA silencing, and therefore influence cellular function. Others (Lennon et al., 2010; Desai et al., 2013) have successfully used DNA based MBs in living cell studies even though it has been reported that DNA based MBs can be degraded and give false positive signals, particularly if the MBs are going into the nucleus (Chen et al., 2007).

From the review above, it is clear that the usage of MB have several advantages over GFP. For instance, cloning, genome incorporation and/or genomic gene replacements are not needed. Furthermore, MB allows for endogenous transcripts to be measured. As opposed to quantitative reverse transcription polymerase chain reaction (qRT-PCR), MB technology is non-destructive allowing for time course studies on the same cell population or culture. However, MBs needs to be developed and tested for each specific gene and as opposed to qRT-PCR methods, there are relatively few MB that has been described to work inside living cells. The aim of this work was to demonstrate the usefulness of MBs to detect dynamic changes in gene expression during differentiation of stem cells into the neural lineage. We designed and used MBs toward *OCT4, SOX2* (Ilieva and Dufva, 2013)*, NeuN, MAP2, Nestin*, and tyrosine hydroxylase (*TH*) mRNAs to track neural stem cell progression from neuronal progenitors, via mature neurons, into highly specialized neurons. Time lapse imaging of growing and differentiating cells allowed us to determine the differentiation status of each cell in the population throughout a 7-day experiment.

## Materials and methods

### Cell culture and media

LUHMES (Lund human mesencephalic cell line, ATCC, CRL-2927) are a subclone of the tetracycline-controlled, v-myc-overexpressing human mesencephalic-derived cell line MESC2.10 immortalized with a LINX v-myc retroviral vector, as described by Lotharius et al. (2002). These cells possess robust dopaminergic characteristics (Lotharius et al., 2005). Undifferentiated cells were cultured and expanded in cell culture flasks pre-coated with Geltrex®, which is a reduced growth factor basement membrane extract purified from murine Engelbreth-Holm-Swarm tumor (Invitrogen). The flasks were coated by incubating for 1 h at 37°C with Geltrex® diluted 1:100 in phosphate buffered saline (PBS). Cultures were maintained in growth medium (GM), consisting of advanced DMEM/F12 (Sigma), 1× N2 supplement (Gibco), 2 mM L-glutamine, 1% penicillin/streptomycin, and 40 ng/ml recombinant basic fibroblast growth factor (bFGF; Invitrogen). Cells were maintained at 37°C in a humidified atmosphere with 5% CO_2_.

HeLa Tet-On® Advanced cells (631155, Clontech) were cultured in DMEM/F12 supplemented with 10% fetal bovine serum (FBS), penicillin 100 U/ml and streptomycin 100 μg/ml.

### Differentiation of LUHMES

The differentiation process was initiated by adding differentiation medium (DM) consisting of advanced DMEM/F12, 1x N2 supplement, 2 mM L-glutamine, 1 mM dbcAMP (Sigma), 1 μg/ml tetracycline (Sigma), and 2 ng/ml recombinant human glial cell-derived neurotrophic factor (GDNF; R&D Systems).

### Design of molecular beacons

mRNA-targeting MBs were designed using Beacon Designer 7.9 (Premier Biosoft). The target sequence for each MB was analyzed using Human Genome BLAST (http://blast.ncbi.nlm.nih.gov/Blast.cgi) to minimize the risk of non-specific binding to unrelated mRNA. Regions not satisfying both the *E*-value and the percentage identity criteria (the percentage of identical bases between query and subject sequence in an alignment, default = 98 for the human genome) were avoided.

It is important to design the MB in an area of the target with minimal secondary structure formation. This helps prevent the template from preferentially annealing to itself faster than to the MB. The program identified and avoided template sequence predicted to give rise to significant secondary structures.

The parameters for MB design were set to ensure that the search succeeded in finding the best possible sequence. The length of the MBs was between 18 and 30 bp. The melting temperature (*T*_m_) values of the probes were calculated using the nearest neighbor thermodynamic calculation with Santa Lucia values (SantaLucia, 1998). The parameters were set as follows: hairpin maximum d*G* (the free energy of the most stable alternate hairpin that is acceptable) 4-kcal/mol; self-dimer maximum d*G* (the free energy of the most stable self-dimer that is acceptable) 7-kcal/mol; cross-dimer maximum d*G* (the free energy of the most stable cross-dimer that is acceptable in a multiplex reaction) 7-kcal/mol; run/repeat maximum 5 bp/dinucleotide probes with single (e.g., AAAAA) runs or dinucleotide (e.g., ATATATATAT) repeats of length greater than the specified value were discarded. After selecting a probe sequence, two complementary arm sequences were added, one on each side of the probe sequence. The stem region of the MBs was 5 bp long, with a CG content of 80%.

All calculations performed with this program used the following default conditions: temperature for beacon free energy calculation 55°C, monovalent ion concentration 100 mM, free Mg^2+^ concentration 3 mM, and target concentration 250 nM.

For the MB probe search, the quality of the designed probe is displayed as “best” (rating greater or equal to 75), “good” (rating between 74 and 50), “poor” (rating below 50), or “not found.” Only MBs with a “best” rating were chosen for synthesis and further evaluation. MBs were synthesized with a 2′-O-methyl RNA backbone, Cy3 molecule attached to the 5'-end, and black hole quencher 2 (BHQ-2) attached to the 3′-end (Eurofins MWG Operon). The sequences of the MBs used in the study are listed in Table 1. MBs were diluted in RNase/DNase free dH_2_O to yield stock concentrations of 100 μM, and stored at −20°C.

**Table 1:** **Molecular beacon sequences**.

**Molecular beacon**	**Sequence**	**GenBank numbers**
*Nestin*	CGCUCUCUCACUACCUCCACAUCCUUGAGCG	NM_006617
*NeuN*	CGCUCUCCCAUUCAGCUUCUCCCGGAGCG	NM_001082575.1
*MAP2*	CGCUCGUUGUCUCUGGCUGAGAAACUAAGAGCG	NM_002374.3
*TH*	CGCUCACACCUUCACAGCUCGGGAGAGCG	NM_199292.2
*OCT4*	CGCUCUCAUUCACCCAUUCCCUGUUGAGCG	NM_203289
*SOX2*	CGCUCCGCCGCCGAUGAUUGUUAUUAUGAGCG	NM_003106.3

### Molecular beacon *in vitro* characterization

Each MB was incubated in 1x PBS at final concentration 0.5 μM. The temperature was ramped from 20 to 90°C with steps of 0.3°C and the fluorescence was measured at every step after 5 min on Chromo4 Real-Time Detection system (BioRad). The melting temperature for each beacon was calculated using the software OligoAnalyzer (http://eu.idtdna.com/analyzer/Applications/OligoAnalyzer/27) and experimentally estimated from the melting curve (data not shown). The maximal signal intensity and signal to noise ratio of 2′O-methyl molecular beacons (MBs) were tested against complementary DNA targets. 0.5 μM of the respective MB and equal concentration of the respective complementary target in 1x PBS was incubated at 37°C. The fluorescence emission was inspected every 5 s for the first 2 min, then every 2 min for 10 min and finally every 15 min for 3 h.

### Toxin-based membrane permeabilization

MBs were introduced into the cytoplasm of living cells using toxin-based membrane permeabilization using streptolysin-O (SLO, Sigma), which is a bacterial exotoxin that reversibly forms pores in the cell surface (Chen et al., 2011). SLO at a concentration 1 μg/ml was activated with the reducing agent tris(2-carboxyethyl)phosphine hydrochloride solution (TCEP, Sigma), at a final concentration 5 mM in Dulbecco's PBS (DPBS) for at least 30 min at 37°C. Cells were washed with DPBS without Ca^2+^ and Mg^2+^, trypsinized for 3 min at 37°C, and collected by centrifugation for 5 min at 190 × g. The activated SLO was diluted in serum free medium (Opti-MEM) to concentrations between 1 and 800 ng/ml, and mixed with MB (2 μM final concentration). Cells (1 × 10^5^) were incubated with the SLO/MB mixture in a final volume of 100 μl in Opti-MEM for approximately 15 min. Afterwards, the permeabilized cells were resealed by washing in DPBS containing Ca^2+^ and Mg^2+^, collected by centrifugation for 5 min at 190 × g, and plated in 12-well plates pre-coated with Geltrex® and containing GM. The differentiation process was started 24 h after plating by exchanging GM for DM.

HeLa cells were transfected as a monolayer with 230 ng/ml (17 U/ml) activated SLO in Opti-MEM. Cells (1 × 10^5^) were washed three times with pre-warmed DPBS and incubated with toxin and MB (2 μM final concentration) in 200 μl Opti-MEM for 15 min. Cells were washed three times with DPBS containing Ca^2+^ and Mg^2+^ and were kept in growth medium.

### Detection of cellular viability

Cellular viability was detected using calcein-propidium iodide staining. Twenty-four hours after SLO treatment without MB medium from each well was carefully removed and cells were incubated for 30 min with 3 μM calcein AM (live cell dye) and 2.5 μM propidium iodide (dead cell dye) diluted in warm 1× DPBS without Ca^2+^ and Mg^2+^.

### Imaging and image analysis

Phase contrast and fluorescent images were acquired using a Carl Zeiss Axio Vision 4.8.2 equipped with ApoTome Imaging system, 40×/0.75 Plan-Neofluar objective, HXP lamp, and a Zeiss Axiocam MRm B/W camera. The same exposure time and filter set (43 HE Ds Red 538–570 nm) were used for all experiments. Single-cell image analysis was performed using ImageJ software (http://rsb.info.nih.gov/ij/). A region of interest (ROI) was drawn around the cells and the total fluorescent intensity (FI) measured. The background fluorescence was detected by drawing a ROI in an area outside the cell of interest, and the total FI measured. In ImageJ, the total fluorescence intensity is reported as integrated density (ID), which is the sum of the values of the pixels in the selection. The background measurement of FI was subtracted from the cellular measurement (Chen et al., 2011). Cells expressing the gene of interest were calculated as the number of cells with positive fluorescent signal emitted from each specific MB per 100 counted cells.

### Quantitative polymerase chain reaction

Total RNA was isolated from cultured cells using the RNeasy Mini Kit (Qiagen). Cells were lysed directly on the dish. The lysates were collected and purified according to the manufacturer's instructions. Single-stranded cDNA was prepared from total RNA using random RT primers under standard conditions using MultiScribe Reverse Transcriptase (Applied Biosystems). The cDNA from each sample was diluted and used for real-time PCR analysis for quantification of neuronal marker expression. TaqMan assays (Invitrogen) for target genes were used as follows: *OCT4* (ID Hs04260367_gH), *SOX2* (ID Hs01053049_s1), *Nestin* (ID Hs00707120_s1), *MAP2* (ID Hs00258900_m1), *NeuN* (RBFOX3 ID Hs01370653_m1), *TH* (ID Hs00165941_m1) with GAPDH (ID Hs03929097_g1) as an internal positive control. PCR amplifications were performed in duplicate using the Chromo4 Real-Time Detection system (BioRad) at 95°C for 10 s, followed by 40 cycles of 95°C for 5 s and 60°C for 30 s. To quantify the relative expression of each gene, the *C*_t_ (threshold cycle) values were normalized to the *C*_t_ value of *GAPDH* [e.g., ΔC_t_= C_t_(target)—*C*_t_(*GAPDH*)]. All experiments included negative controls containing no cDNA template.

### Statistical analysis

Results are expressed as mean ± standard error of the mean (s.e.m). qPCR was analyzed using a Mann-Whitney test (*n* = 5). Analyses were performed using XLSTAT Version 2013.5.07 (Addinsoft).

### Diffusion of MB out of the cells

The adherent cell line HeLa was transfected with MB toward *TH* using the SLO. This particular MB did not contain a quencher and was thus always fluorescent even when the MBs are in the closed state. The pores were closed after transfection by washing three times with DPBS containing Ca^2+^ and Mg^2+^, and incubated for 1 h to let cells recover in growth medium. Hybridization was not expected because *TH* is not expressed in this particular cell line. The cells were fluorescent as expected and had their normal morphology (data not shown) after transfection. At this point the cells were again treated with SLO reagent to re-open the pores and the fluorescent decay from the cells was monitored by time laps microscopy for 20 min.

## Results

### Introduction of MBs into cells

**Figure 1:**
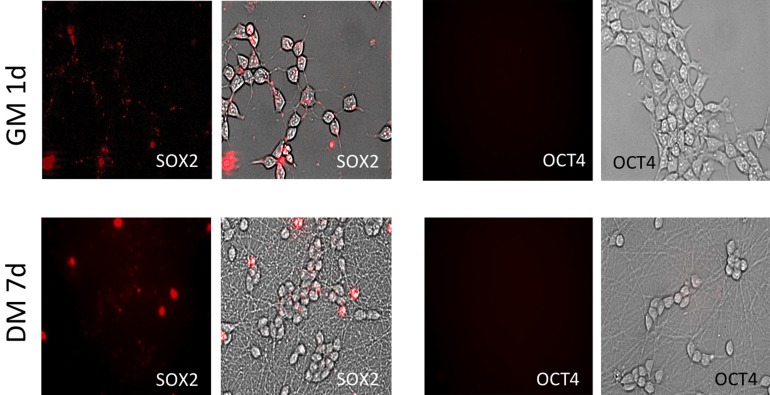
**Fluorescent images of cells transfected with MBs targeting stem cell markers *SOX2* and *OCT4*.** High transfection eficiency was detected using SLO and ~95% of cells shows to be positive for *SOX2* MB after 1 d in GM. Relatively the same number of SOX2 positive cells was detected after 7 d differentiation but with significant depletion in fluorescent intensity. Signal from *OCT4* MBs was not detect during the whole experimental period.

Cell viability and transfection efficiency were investigated as a function of SLO concentration ranging from 1 ng/mL (corresponding to 0.07 U/mL) to 800 ng/mL (corresponding to 59.7 U/mL). Undifferentiated LUHMES cells that express *SOX2* but not *OCT4* mRNA (Ilieva and Dufva, 2013) were used in the optimization. An SLO concentration of 17 U/mL was determined to be the optimal concentration, with near 95% of the cells showing signal from MB targeting *SOX2* 24 h post-transfection (Figure 1), and with >95% cell viability after SLO treatment (Supplementary Figure S1A). *OCT4* mRNA was not expressed (Figure 1). Moreover, cells showed no defects in growth or differentiation and they exhibited the same morphology as non-transfected cells (untransfected cells are shown in Supplementary Figure S1B, and transfected cells in Figure 4). An SLO concentration of 17 U/mL (230 ng/mL) was therefore used in the further experiments.

For analytical purposes, it is of utmost importance to have an estimate of the number of MB that enters cells during transfection. The SLO process opens pores during the transfection and then the MB enters the cells by diffusion. This transfection mechanism will yield a maximum intracellular concentration of MB that is equal to the MB concentration outside the cells (in this case 2 μM).

The time it takes to reach maximum concentration inside cells was measured experimentally and estimated theoretically in order to ensure that this maximum concentration could be reached during the 15-minute transfection.

**Figure 2:**
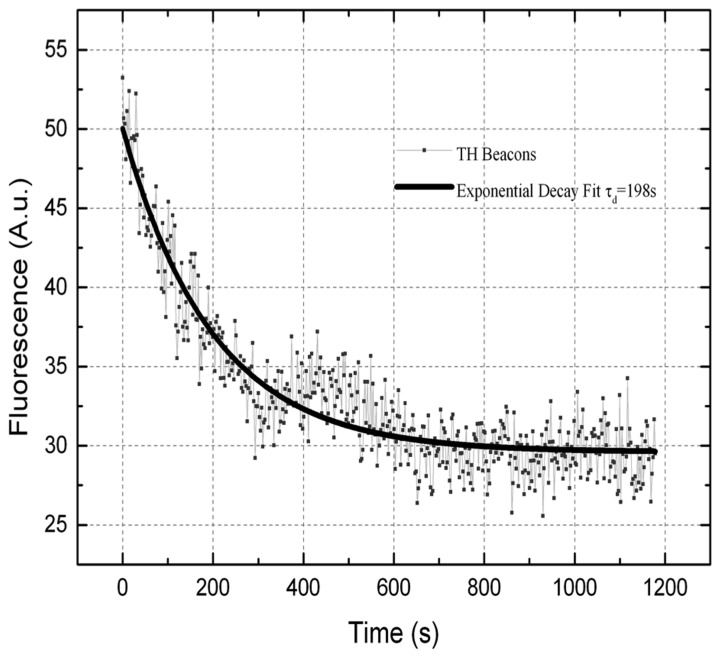
**Fluorescence emission decay as a function of time for a representative HeLa cell after the second treatment with SLO.** The resulting time constant is τ_exp_ = 198 s.

Direct experimental tracking the fluorescence during transfection is difficult due to the high background from MBs in the medium. Instead the decay of fluorescence from cells loaded with MBs lacking a quencher was tracked. It took about 10 min for all MBs to leave the cells (Figure 2), which is less than the 15-min transfection protocol.

**Figure 3:**
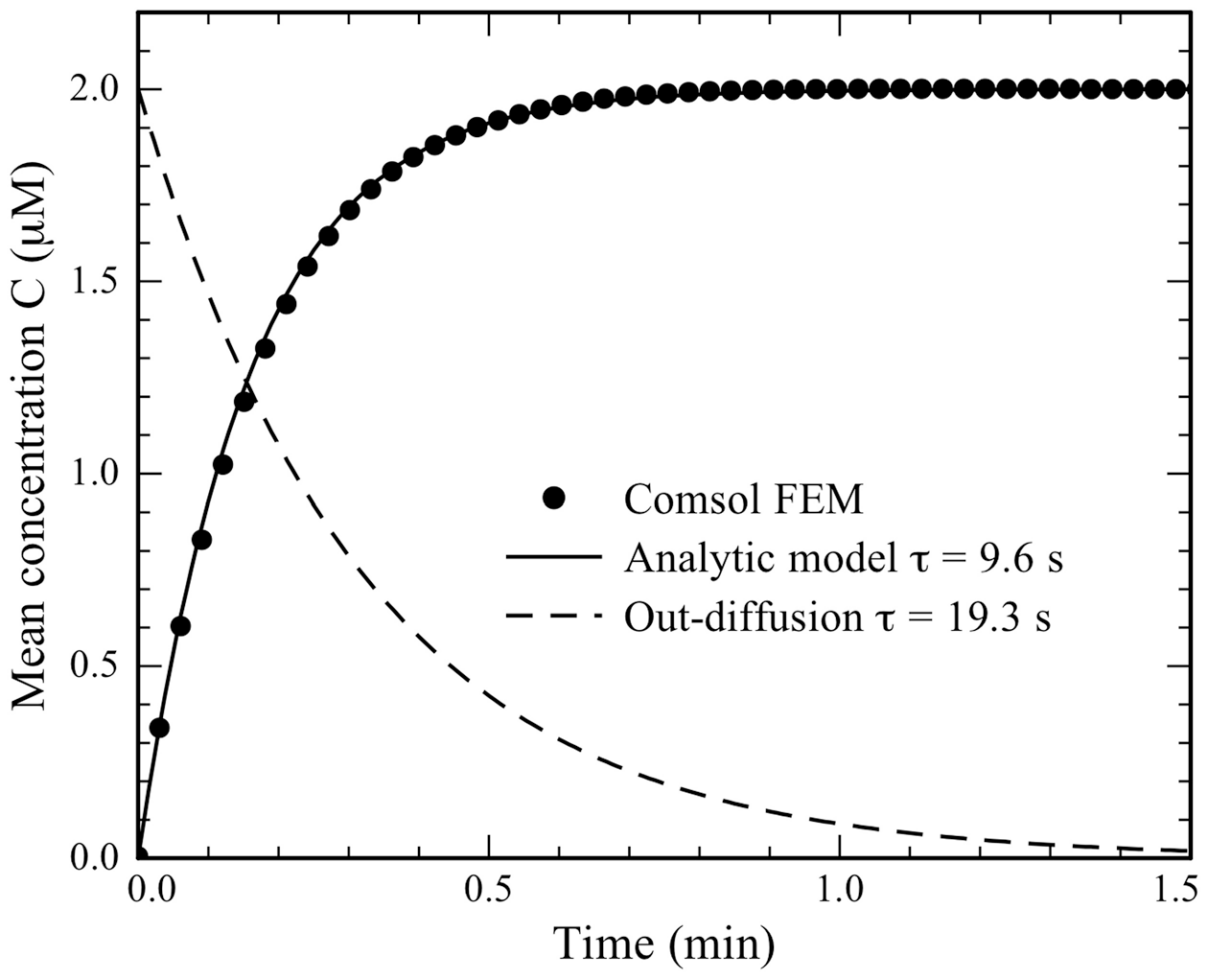
**The average concentration of MBs in the cell as a function of transfection time.** Results from a COMSOL FEM model (dots) are compared to the analytical model (black line) with the time constant τ_c_ = 9.6 s. The average concentration in the outdiffusion experiment calculated from the analytic model is shown as the dashed curve using the time constant τ_o_ = 19.3 s.

A finite element model (FEM) and an analytical model (see Supplementary information for details) of diffusion from the medium, through the pores and into the cells agree (Figure 3) that it takes approximately 0.5 min to reach maximum concentration in the cells. This is faster than the experimental result, but the models are very sensitive to the MB diffusivity, pore size, and number of pores, parameters that are difficult to estimate correctly (see Discussions).

Therefore it is likely that there are about 60,000 MB molecules inside the cells after transfection (see Supplementary information for example calculations). However, the number of MBs in the cells is heavily dependent on the cytoplasmic volume compared to the nuclear volume. A cytoplasmic volume of 20%, the cells will each contain in 120,000 MBs after transfection while a cytoplasmic volume of 10%, the cells will each contain 60,000 MBs after transfection. Bustin (2000) found that there are about 10^8^ mRNA copies of GAPDH per microgram of total RNA. Assuming that there is about 10 pg total RNA per cell (Bustin, 2000), there would be about 1000 GAPDH mRNA copies per cell, indicating that there is at least 50-fold more MB than possible target.

### Gene expression of stem cell and neuronal markers detected by MBs

MBs targeting mRNAs specific for stem cell (*OCT4, SOX2*) neural progenitors (*Nestin*), mature neurons (*NeuN* and *MAP2*), and highly specialized neurons (*TH*) were designed (material and methods). The function of the respective MB was evaluated by hybridization to their respective perfect match target in 1 X PBS. The fluorescence of the MB in hybridized state with the complementary target vs. fluorescence of MB in unhybridized state (signal to noise ratio) and maximal signal intensity of hybridized MB were evaluated. The S/N ratio varied greatly and only MB-OCT4 had low S/N ratio (*OCT*4 = 2, *SOX*2 = 23, *TH* = 47, *MAP*2 = 10, *nestin* = 25, and *N*euN = 13). However, the MB-OCT4 probe has previously successfully been used to measure OCT4 expression inside neurosphere formation of LUHMES cells (Ilieva and Dufva, 2013). The maximum signals obtained from the respective beacons also varied (in relative fluorescent units: *OCT*4 = 7, *SOX*2 = 15, *TH* = 17, *MAP*2 = 19, *N*estin = 29, and *N*euN = 10). Moreover, unhybridized MBs had a measured melting point above 40°C in 1XPBS (data not shown) which verified that the MBs were functionally closed at physiological conditions.

**Figure 4:**
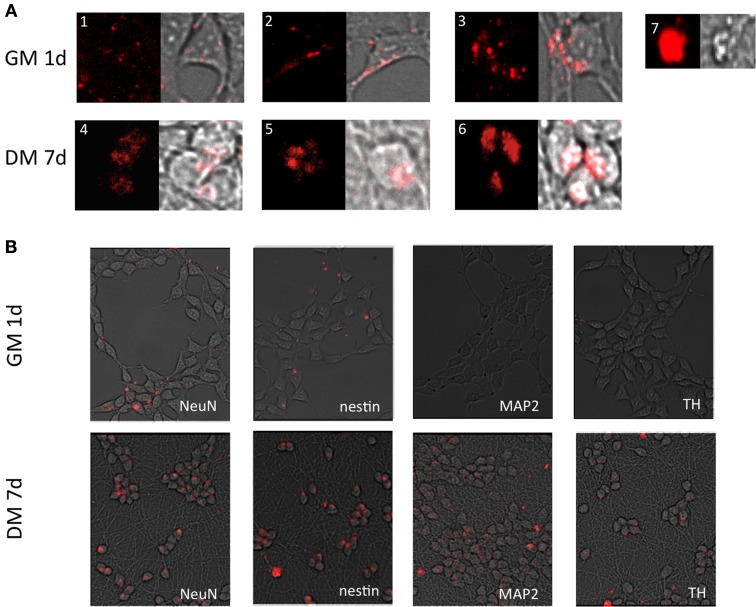
**Fluorescent images of cells transfected with MBs. (A).** Different MB signal patterns observed in LUHMES cells grown in GM 1 day after transfection (**A1–3**) and 7 days post induction of differentiation (**A4–6**). (**A7**) shows large bright fluorescent clusters originating from apoptotic cells (artifact). These cells were excluded from calculations of the number of positive cells and signal intensity. **(B)** Signals (red) from the respective MBs—*SOX2, nestin, NeuN, MAP2, TH* from a population of cells. Please see Supplementary information S2 outline of the experiments.

The respective MBs targeting stem cell and neuronal markers were delivered into embryonic midbrain derived cell line (LUHMES). The patterns of fluorescence signal emitted from cells varied (Figure 4), and included small dotted signals (Figure 4A1), more compact punctate signals (Figure 4A2), or widespread cytoplasmic cluster-like fluorescence (Figure 4A3). The last pattern was more often observed in differentiated cells (Figures 4A4–6), due to the compact cell body and tight perinuclear cytoplasmic organization. Non-differentiated cells showed signals in the lamellipodia and cell body space (Figure 4A2), while differentiated cells showed only cell body-localized MB signals (Figures 4A4–6). All signal categories were included in the analysis when calculating the percentage of positive cells (see below). Large bright signals were interpreted as false positives, as these cells displayed dead cell morphology—reduced volume of the cell body, irregular surface, often detached from the growth matrix (Figure 4A7). Moreover, cells showing this morphology showed propidium iodide staining which is an indicator for dead cells (data not shown). Therefore, these false positive signals were excluded from the analysis.

**Figure 5:**
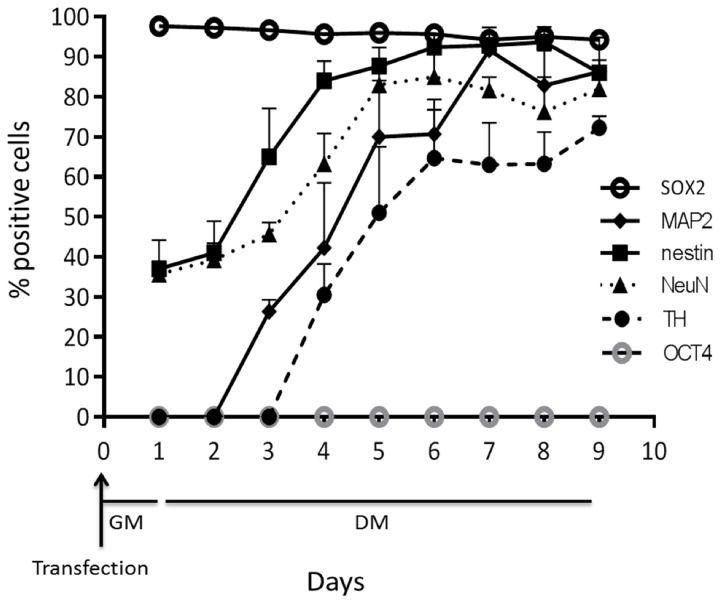
**The number of positive cells from MBs targeting OCT4, SOX2, Nestin, NeuN, MAP2, and TH over time.** Results from three independent experiments are presented as mean ± standard error of the mean (s.e.m).

**Figure 6:**
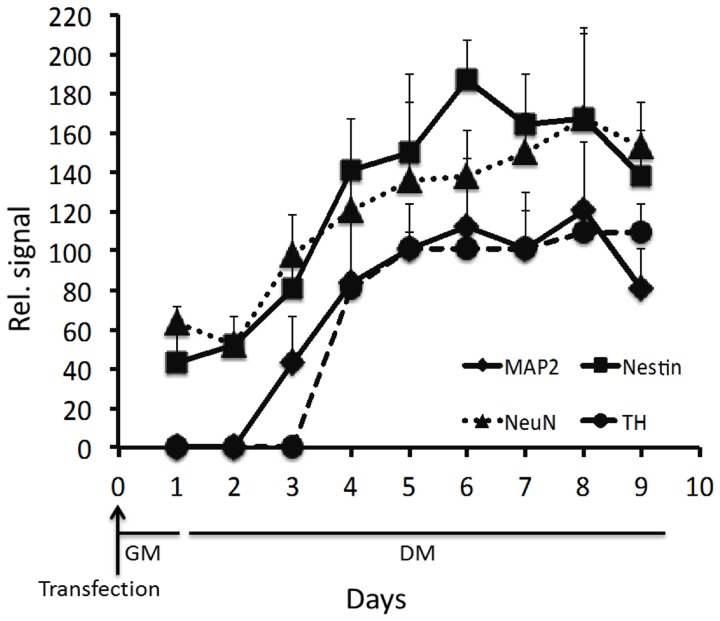
**Relative signal for the respective MB.** The fluorescence was measured 1 day after transfection, and subsequently every day for 8 days of differentiation. Results from three independent experiments are presented as mean ± standard error of the mean (s.e.m).

Ninety-five percent of cells were positive for *SOX2* mRNA 1d (1 day) post-transfection (Figures 1, 5). After 8d differentiation, the same number of the cells was positive for *SOX2* mRNA (Figures 1, 5). By contrast *OCT4* mRNA was not detected during the whole experiment in any of the cells (Figures 1, 5). The relatively poor ability of measuring down regulation of *SOX2* mRNA using the described *SOX2* beacon has been discussed elsewhere (Ilieva and Dufva, 2013) and explains why we do not see a large drop in the number of *SOX2* positive cells. *Nestin*, an intermediate filament protein expressed in dividing cells during the early stages of development of the CNS, and nuclear protein antigen (*NeuN*), were also detected 1d after transfection in about 35% of cells (Figures 4B, 5). In contrast, no *MAP2* and *TH* expression was detected in cells 1d post transfection (Figures 4B, 5). Expression of *MAP2* was detected in 30% of cells 2d post induction of differentiation (Figure 5), while the first *TH*-positive cells appeared 3d post induction (Figure 5). The number of cells expressing neuronal markers reached their maximum at different time points as follows: 85% *Nestin* and *NeuN*-positive cells 5d, 85% *MAP2*-positive cells 6d, and 70% *TH*-positive cells 8d post induction of differentiation, respectively. Signals from neuronal markers *NeuN* and *TH* targeting MBs were not detected in the control non-neural cell line—adipose-derived stem cells (ASC) (Supplementary Figure S3). The intensity of the nestin-MB signal increased about two-fold compared to the point-of-induction level. The maximum increase in *Nestin*-MB signal was observed 5d post induction. A slight decrease in the signal was detected 8d after induction (Figure 6). The maximal signal of *NeuN* was observed 7d post induction (Figure 5). The *MAP2*-MB signal maximized 5d post induction. *TH*-MB signal plateaued 4d post induction. *MAP2*-MB signal intensity decreased slightly 8d post induction, while *TH* levels remained constant (Figure 6).

### Correlation between qRT-PCR and MB signal intensities

**Figure 7:**
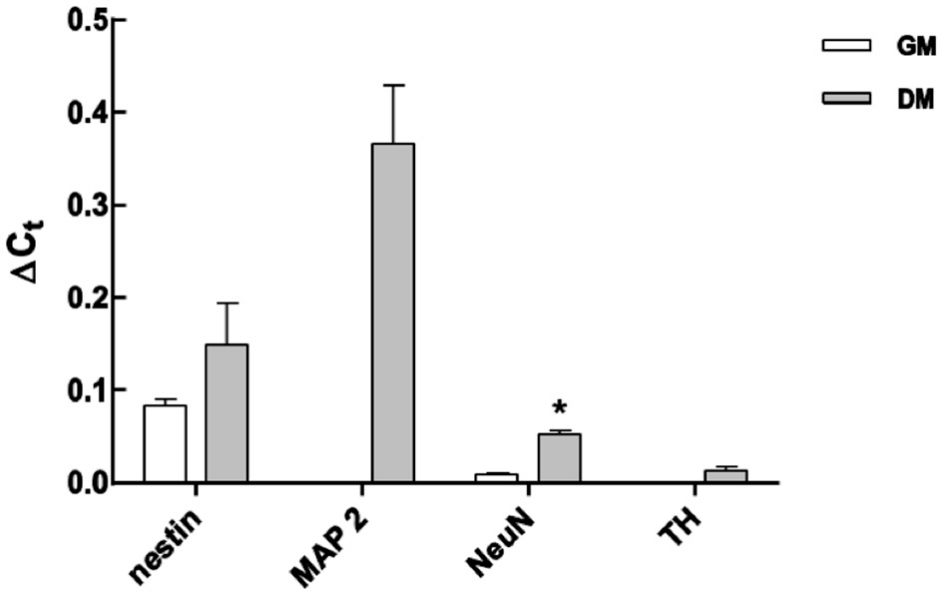
**Analysis of mRNA expression levels of neuronal markers using qRT-PCR in non-differentiated and differentiated LUHMES.** Expression was normalized to GAPDH mRNA expression. Data from five independent experiments are expressed as mean ± s.e.m. ^*^P < 0.05.

To corroborate the results using different MBs, qRT-PCR was used to measure the respective mRNA expression before and 7 days after induction of differentiation. *GAPDH* was used as an internal positive control. Expression of *Nestin* and *NeuN* was detected in non-differentiated cells, and it significantly increased after 7 days of differentiation (Figure 7). In contrast, expression of the neuronal markers *MAP2* and *TH* was not detected in non-differentiated cells, but was later expressed, corroborating the accuracy of the measurements made using MBs (Figures 3–5).

## Discussion

One significant advantage of *in situ* methodologies, and particularly MB technology, is the ability to gather spatial and temporal data on biological phenomena of interest. There appears to be single cell heterogeneity during neuronal differentiation of a population of LUHMES. There were about 35% *Nestin* and *NeuN* mRNA positive cells in non-differentiating cells. In contrast, 95% of the cells showed expression of *SOX2* mRNA, which indicates that there is a transfected population of cells that is not expressing *Nestin* or *NeuN*. Alternatively, this subpopulation of cells express *Nestin* and *NeuN* in too low concentrations to be detected by the respective MB. *Nestin* is a nerve stem cell marker found in proliferating cells which expression is normally decreased during differentiation. However, *Nestin* was expressed in equal level in non-differentiated LUHMES cells and a relatively pure population of differentiated LUHMES cells (Scholz et al., 2011). Furthermore, *Nestin* was not down regulated during differentiation in some clones of human nerve stem cells derived from ventral mesencephalon (Ramos-Moreno et al., 2012). The data presented here using MBs (Figure 6) and qRT-PCR (Figure 7) supports that *Nestin* is not down regulated to large extent during differentiation of LUHMES cells.

The heterogeneity of LUHMES cells is supported by previous work where it was noted that some cells continue to proliferate to some extent during differentiation (Scholz et al., 2011). These proliferating cells subsequently start to differentiate. Re-plating of differentiating LUHMES cells at 2d results in 85–95% cell with neuronal phenotype (Scholz et al., 2011) and without re-plating >80% of the cells become *TH* positive (Lotharius et al., 2002) indicating that the re-plating procedure removes proliferating cells (Scholz et al., 2011). In the present study re-plating was not used which resulted in about 70% of the cells expressing *TH* mRNA according to MB data.

Furthermore, here we report that the *TH* mRNA is expressed at 3d after induction of differentiation with stable expression from 4d. It was previously reported that TH protein expression in LUHMES was detected at 4d (3d was not tested) after induction of differentiation (Lotharius et al., 2002). *NeuN* protein expression increase during differentiation of LUHMES (Scholz et al., 2011) with maximum expression levels at 5d post induction of differentiation. The present study indicates that the *NeuN* mRNA reached highest expression level at d7. Scholz et al. (2011) showed that about 75% of the differentiating cells were positive for NeuN protein at d5 while the present study showed that *NeuN* mRNA was expressed in about 80% of the cells at d5. At day 0, however, we have conflicting results where the present study shows that about 30% of the cells are positive for *NeuN* mRNA while Scholz et al. (2011) showed that there was <1% NeuN protein positive cells. It is, however, noteworthy that all these studies typically do not show expression levels on the per day basis as reported here (Figures 5, 6). The reason is likely that destructive analysis requires large number of parallel experiments to gather information over time, which is costly and cumbersome. The clear advantage of MBs is that they, like GFP technology, enable non-destructive analysis of gene expression.

The MBs used in this study was made of 2-O-methyl RNA, which avoids degradation of the MB with a resulting false positive signal. The mRNA signals in this study only appeared in the cytoplasm indicating the fluorescent MB was reacting with mRNA species. If MB where present in the nucleus is unknown but no nucleic signal were observed indicating that MB were stable in the nucleus and did not bind unspecifically to DNA. The specificity of each beacon in terms of hybridization to its intended target inside cells is somewhat circumstantial but a few lines of evidence indicate that these MBs are indeed specific. The MBs reacted *in vitro* (test tubes) only with the perfect matched target and not to other synthetic targets (data not shown). The beacons toward the neuronal markers did not result in signals in all the transfected cells 1 day after transfection while fluorescent signals from SOX2 MB transfected cells were detected in 95% of the cells at the same time point. Moreover, the signal from *OCT4* MB was not detected throughout the experiment, suggesting that the increase in the number of cells expressing neuronal markers was due to binding to the respective complementary target mRNA and not time depended unspecific binding to mitochondria (Rhee and Bao, 2010) or nuclease digestion. Finally, the control non-neural cell line ASC did not show any signal from *NeuN* and *TH* MBs in the time where both neuronal markers are already expressed in LUHMES, proving target specificity of described MBs. It also suggest that modified MBs in general is not degraded inside the living cells (see also Oct4 data in Figure 5). The MB signal for the neuronal markers follows the same trend as q-RT-PCR data (Figures 5, 6) and the expression pattern follows closely other reports (see above). Furthermore, probe-target hybridization did not appear to have any measurable effect on cell physiology including gene expression, self-renewal, or differentiation (supplementary Figures 1, 3).

The results here demonstrate that about 60000 MB loaded per cell during the transfection. Transfection of *SOX2*-MB resulted in ~95% cell giving signals (Figures 4, 5) suggesting that the vast majority of the cells received MBs. The amount of MB transfected into the cells may, however, vary as a function of number of pores formed and the speed, which they are formed. Pore forming times between 10 and 300 s have been recorded (Niedermeyer, 1985 and Palmer et al., 1998). There can therefore be a delay before MB can enter the cells, which was not considered in the simulation and analytical solutions. The pores exhibit a size distribution and 30 nm is considered an upper limit for the diameter (Palmer et al., 1998). It is therefore possible that smaller pores are formed, which would contribute to the explanation of why the analytical (Figure 3) determined and measured transport (Figure 2) did not overlap. It should be noted that differentiating LUHMES leaves the cell cycle and decreases in signal intensity due to MB dilution should therefore not be a large problem. At least a few cell divisions are allowed before any dilution effect can be expected (Ilieva and Dufva, 2013). As estimated above, there is on average a 50-fold excess of MBs to GAPDH molecules in the respective transfected cells. For lower expressed genes the excess is even larger. With such an excess of MBs, it is likely that there are enough MBs in the majority of the cells to measure the desired mRNA even if some variance in transfection is expected.

Lipid and dendrimer-based oligonucleotide delivery methods, have often failed to deliver MBs into cells. MB-transfection agent complexes do not always dissociate efficiently once internalized, leading to brightly fluorescing punctate aggregates that interfere with fluorescent measurements. Furthermore, MB-transfection agent complexes can often lead to entrapment and degradation of the probes within endosome/lysosome compartments, thus increasing background signal. The transfection efficiency of neurons is particularly limited (Karra and Dahm, 2010), and indeed LUHMES represent a particularly challenging system, since they cannot be transfected using standard methods, such as lipo- or nucleofection. One routinely used method for direct introduction of MBs into the cytoplasmic compartment of living cells is toxin-based membrane permeabilization using streptolysin-O (SLO), as used in this study. Although it has been successfully used to deliver MBs into a wide variety of cell types, including human dermal fibroblasts, stem cells, and cancer cells (Chen et al., 2011), SLO is not a popular method for neuronal transfection. Here we show that the SLO-membrane permeabilization delivery method is a rapid, efficient, and gentle method for the delivery of MBs into neuronal cells. Cells viability was high and the transfection efficiency nearly 100%.

In conclusion, MBs offer significant advantages over traditional techniques because they allow for real-time imaging of individual cells and preserve spatial information. In contrast to PCR-based assays, which collect data from the differentiating population as a whole, MBs accurately measure expression of genes of interest on a single cell level. MBs can be used to repeatedly assess gene expression in the same cell population over time, which is not possible using the destructive methods. Moreover, they overcome the need to lyse cells for qRT-PCR, or fix cells for molecular imaging techniques such FISH. Time-consuming and technically challenging genetic manipulations are furthermore not required.

## Author contributions

Conceived and designed the experiments: Mirolyuba Ilieva, Paolo Della Vedova, Martin Dufva, Ole Hansen. Performed the experiments: Mirolyuba Ilieva, Paolo Della Vedova. Analyzed the data: Mirolyuba Ilieva, Paolo Della Vedova, Martin Dufva, Ole Hansen. Modeling of MB loading into cells: Paolo Della Vedova, Ole Hansen. Wrote the manuscript: Mirolyuba Ilieva, Martin Dufva. Directed and supervised the project Martin Dufva, Ole Hansen.

## Conflict of interest statement

The authors declare that the research was conducted in the absence of any commercial or financial relationships that could be construed as a potential conflict of interest.
