# Supplementary material for: Tracking neuronal marker expression inside living differentiating cells using molecular beacons
Source: Front Cell Neurosci. 2013 Dec 19;7:266. doi: 10.3389/fncel.2013.00266 (PMC3883158; doi:10.3389/fncel.2013.00266)
Supplement: Supplementary file 1 [file DataSheet1.PDF]

Tracking neuronal marker expression inside living differentiating cells using molecular beacons

Mirolyuba Ilieva[1], Paolo Della Vedova[1], Ole Hansen[1,2], Martin Dufva[1*]

[1]Department of Micro- and Nanotechnology, Technical University of Denmark, DTU Nanotech, Building 345E, DK-2800 Kgs. Lyngby, Denmark. [2]CINF - Center for Individual Nanoparticle Functionality, Technical University of Denmark, DK-2800 Kgs. Lyngby, Denmark. **Corresponding Author: Martin Dufva, Department of Micro- and Nanotechnology, Technical University of Denmark, Ørsteds Plads 345 Ø, building 344, 2860 Kgs. Lyngby, Tel. +45 45 25 63 24,, Fax. +45 45 88 77 62, martin.dufva@nanotech.dtu.dk , www.nanotech.dtu.dk*

<u>Supplementary information:</u>

Detection of cellular viability

Cellular viability was detected using calcein-propidium iodide staining. Medium from each well was carefully removed and cells were incubated for 30 min with 3 µM calcein AM (live cell dye) and 2.5 µM propidium iodide (dead cell dye) diluted in warm 1x DPBS without Ca^{2+} and Mg^{2+}.

S1

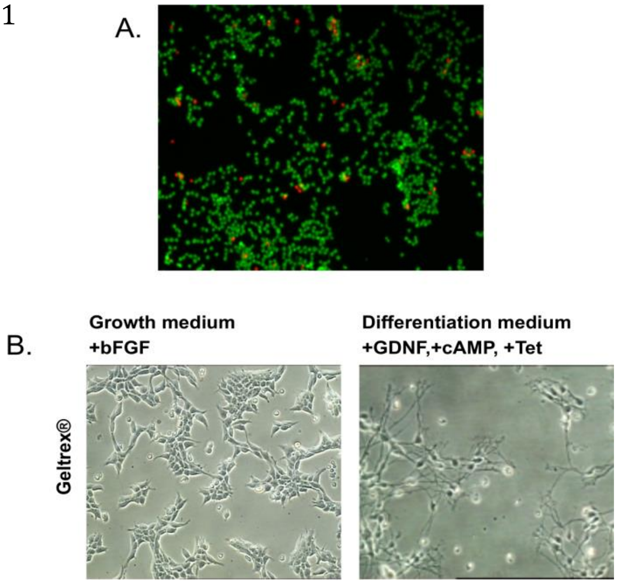

Figure S1. (A) Cells treated with 17 U/ml SLO and stained with calcein/PI for detection of cell viability after toxin-based membrane permeabilization. Live cells are stained in green, dead cells are stained in red. (B) Morphology of non-transfected LUHMES on Geltrex® in GM (left) and DM (right).

Schematic representation of the experimental design

Cells were transfected with MBs using toxin-based membrane permeabilization. Cells were left for 24 h recovery in growth medium (GM). On the day 0 phase

contrast and fluorescent images were acquired and differentiation process was induced by switching GM to differentiation medium (DM). Images were taken every 24 h for eight days.

S2

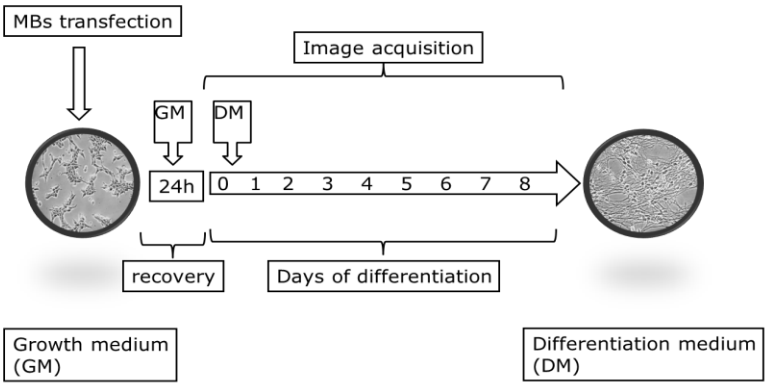

Adipose-derived stem cells (ASC) growth and transfection with MBs targeting for neuronal markers NeuN and TH

ASC isolated from liposuction aspirates were maintained in DMEM/F12 medium with GlutaMax, 10% fetal calf serum and 1 % Penicillin/Streptomycin. MBs targeting NeuN and TH were introduced by SLO based membrane permeabilization. Cells were washed with DPBS without Ca2+ and Mg2+, trypsinized for 5 min at 37°C, and collected by centrifugation for 5 min at 200 x g. Cells (1x105) were incubated with the activated SLO at final concentration 230 ng/ml (17 U/ml) and MB (2 µM final concentration) in a final volume of 100 µl in Opti-MEM for approximately 15 min. Afterwards, the permeabilized ASCs were resealed by washing in DPBS containing Ca2+ and Mg2+, collected by centrifugation for 5 min at 200 x g, and plated in complete growth medium.

S3

A.

B.

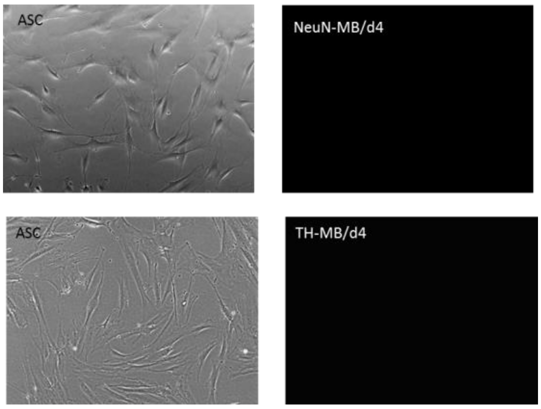

S3. Phase contrast and Fluorescent images of Adipose-derived stem cells (ASC) four days after transfection with MBs targeting for neuronal markers A. NeuN and B. TH.

Modeling of MB loading into cells

The transport of MB's into the cells during transfection is assumed to be a diffusion problem, where MBs diffuse from the bulk medium to pores in the cells that are formed due to the SLO treatment, through the pores and into the cells.
Diffusion problems in dilute systems are governed by Fick's first

$$\mathbf{J} = -D\nabla C, \tag{1}$$

and second laws

$$\frac{\partial C}{\partial t} = -\nabla \cdot \mathbf{J} \cong D\nabla^2 C, \tag{2}$$

where C is the concentration, **J** is the diffusion flux density, D the diffusivity, and t is time.
The diffusivity of the MB's in the medium may be estimated from the fit to experimental diffusivity data for similar DNA structures in (Lukacs et al., 2000)

$$D = 4.9\times10^{-6}\times[\mathbf{bpsize}]^{-0.72}\mathrm{cm}^2/\mathrm{s}, \tag{3}$$

where bpsize is the number of base pairs in the fragment, which for 35 base pairs results in the diffusivity D = 38 μm^2/s. The diffusivity D_c of the MB's in the cytoplasm and the pores is assumed to be lower by a factor of approximately 5 (Lukacs et al., 2000), and thus D_c = 7.6 μm^2/s.
By use of the Stokes-Einstein equation for the diffusivity

$$D = \frac{k_B T}{6\pi\eta a_b}, \tag{4}$$

where η is the viscosity of the medium, k_B is Boltzmann's constant and T the absolute temperature, the effective hydraulic radius a_b of the MB's may be calculated. At 300K and a viscosity of η = 0.85 mPa s the result is a_b = 6.8 nm.
We now model the diffusion problem assuming spherical cells with the radius a = 5 μm and with a cell membrane that is x_m = 10 nm thick. In the membrane N_p = 600 pores (2 pores per μm^2) with a pore radius of a_p =15 nm are opened during transfection (Keyel et al., 2011). The molecular beacons are modeled as solid spheres with the radius a_b, and a C_0 = 2 μM concentration is used in the medium.
The real diffusion problem is a rather complicated 3D problem; however, the different length scales involved in the problem suggest that approximations may be made. Recognizing, that typical diffusion times are $t_d = \lambda^2/(2D)$, where λ is the characteristic length scale, results in diffusion times for the pore regions that are five orders of magnitude lower than diffusion times relevant on the cell scale. Thus a quasi steady-state approximation may be applied to the pore regions. Furthermore, the pores are so far apart that they hardly interact in the diffusion process. As a result, we may estimate the diffusion flux through a single pore by the net flux I that results from 1) quasi steady-state diffusion to a disc of radius a_p- a_b (to compensate for the finite size of the MB's), 2) steady state diffusion through a tube of radius a_p- a_b and length x_m, and 3) quasi steady-state diffusion from a disc of radius a_p- a_b to the cell interior. The first flux is given by $I_1 = 4(a_p- a_b)D\Delta C_1$ (Carslaw and Jaeger, 1959) where ΔC_1 is the concentration difference that drives the flux. The second flux is $I_2 = (\pi(a_p- a_b)^2/ x_m)$

$D_c\Delta C_2$, where the symbols have the same significance as above. The third flux is similar to the first and is given by $I_3 = 4(a_p - a_b)D_c\Delta C_3$. The three fluxes are identical while the sum of concentration differences $\Delta C_1+\Delta C_2+\Delta C_3 = C_0-C_c$ where C_c is the concentration inside the cell, which is considered approximately uniform. As a result the flux through a single pore is

$$I_p = \left(\frac{1}{4(a_p - a_b)D} + \frac{1}{4(a_p - a_b)D_c} + \frac{x_m}{\pi(a_p - a_b)^2 D_c} \right)^{-1} (C_0 - C_c), \quad (5)$$

and the total flux through all pores is of course N_p times larger. Integrating Fick's second Law, Eq. (2), over the volume $V = 4\pi a^3/3$ of the cell yields

$$\frac{dC_c}{dt} = \frac{N_p I_p}{V} \equiv \frac{C_0 - C_c}{\tau_c}, \quad (6)$$

where τ_c is the time constant relevant for the transfection dynamics

$$\tau_c = \frac{V(C_0 - C_c)}{N_p I_p} = \frac{4a^3}{3N_p} \frac{x_m + \frac{\pi}{4}(a_p - a_b)(1 + D_c / D)}{(a_p - a_b)^2 D_c}. \quad (7)$$

The solution to equation (6) is easily obtained

$$C_c = C_0\left(1 - \exp\frac{-t}{\tau_c}\right), \quad (8)$$

and within a few time constants the concentration of molecular beacons in the cells saturates at C_0.

A Finite Element Model (FEM) simulation using COMSOL 4.2 (http://www.comsol.com) was employed to verify the diffusion model. In the FEM model, a cone segment of the spherical cell with exactly one pore was used to represent a $1/N_p$ fraction of the cell volume. The cone region was extended far into the free fluid volume in order to also represent a $1/N_p$ fraction of that.

With the numerical values given the time constant for transfection becomes $\tau_c = 9.6$ s, a value that is very sensitive to e.g. the number and size of the pores. With this time constant the MB concentration will reach the final value after approximately 0.5 min. The relevant time constant for the out-diffusion experiment may be estimated using the same equation, but since the cells in that case were immobilized on a support, we assume that half of the pores are un-accessible and thus that time constant increases by a factor of 2 to $\tau_o = 19.3$ s. As a result the out-diffusion is expected to be complete after approximately 1.5 min.

Calculation of MB concentration inside transfected cells

If it is assumed that the nucleous occupies 80% of the volume we get:

$C = 2\frac{\mu\text{mol}}{(\text{dm})^3}$

$V = \frac{4\pi}{3}r^3 = \frac{4\pi}{3}(5\,\mu\text{m})^3 = 5.2360 \times 10^{-16}\,\text{m}^3$

$V \times 0.2 = 0.2 \times \frac{4\pi}{3}(5\,\mu\text{m})^3 = 1.0472 \times 10^{-16}\,\text{m}^3$

$C \times V \times 0.2 = 2\frac{\mu\text{mol}}{(\text{dm})^3} \times 1.0472 \times 10^{-16}\,\text{m}^3 = 2.0944 \times 10^{-19}\,\text{mol}$

$N = C \times V \times 0.2 \times N_A = 2.0944 \times 10^{-19}\,\text{mol} \times 6.0221367 \times 10^{23}\,\text{mol}$

$1.2613 \times 10^5 = 126130 \simeq 120000$

References

Carslaw HS, Jaeger JC. Conduction of heat in solids. In: London: Oxford University Press 2nd Ed. 1959. p. 215.

Keyel PA, Loultheva L, Roth R, Salter RD, Watkins SC, Yokoyama WM, Heuser J. Streptolysin O clearance through sequestration into blebs that bud passively from the plasma membrane. J Cell Sci 2011;124:2414-23.

Lukacs GL, Haggie P, Seksek O, Lechardeur D, Freedman N, Verkman AS. Size-dependent DNA mobility in cytoplasm and nucleus. J Biol Chem 2000;275:1625-9.
